# Association between low-dose aspirin use and breast cancer recurrence: a Danish nationwide cohort study with up to 23 years of follow-up

**DOI:** 10.1038/s41416-025-03112-3

**Published:** 2025-07-22

**Authors:** Elisabeth Solmunde, Rikke N. Pedersen, Mette Nørgaard, Lene Mellemkjær, Søren Friis, Bent Ejlertsen, Thomas P. Ahern, Deirdre P. Cronin-Fenton

**Affiliations:** 1 Department of Clinical Epidemiology, Department of Clinical Medicine, Aarhus University and Aarhus University Hospital, Aarhus, Denmark; 2 Danish Cancer Institute, Danish Cancer Society, Copenhagen, Denmark; 3 Danish Breast Cancer Group, Department of Clinical Medicine, Rigshospitalet and Copenhagen University Hospital, Copenhagen, Denmark; 4 Department of Surgery, The Robert Larner, M.D. College of Medicine, The University of Vermont, Burlington, VT USA

**Keywords:** Cancer epidemiology, Epidemiology, Breast cancer

## Abstract

**Background:**

The anti-cancer potential of low-dose aspirin in long-term breast cancer (BC) survivors remain unknown. We evaluated the association between low-dose aspirin use and BC recurrence and mortality.

**Methods:**

Women ≥40 years diagnosed with stage I-III BC (1996–2004) were identified from the Danish Breast Cancer Group (DBCG) database and information on aspirin use from the Danish Prescription Registry. We ascertained recurrences from DBCG and via a validated algorithm. We plotted cumulative incidences of recurrence and mortality, accounting for competing risks. Using Cox regression, we estimated hazard ratios (HRs) and 95% confidence intervals (CI), employing landmark analyses at 5-, 10-, and 15-year post-diagnosis.

**Results:**

Among 20,509 BC survivors, 4527 developed recurrence over 232,441 person-years of follow-up. The 20-year cumulative incidence of recurrence was lower in users (17.8%) than nonusers (22.4%), with similar trends among 10-year disease-free survivors (9.9% vs. 12.7%). We observed reduced HRs of recurrence (adjusted HR_5-year_ = 0.80, (95% CI = 0.66-0.98); HR_10-year_ = 0.87 (0.73–1.05); HR_15-year_ = 0.82 (0.57–1.17) in aspirin users, but increased HRs of all-cause mortality (HR_5-year_ = 1.08 (0.96–1.21); HR_10-year_ = 1.09 (0.96–1.24); HR_15-year_ = 1.09 (0.80–1.31).

**Conclusions:**

The reduced recurrence risk in aspirin users may indicate potential anti-cancer effects of aspirin, though the increased risk of death suggests influence by confounding by indication and competing risks.

## Introduction

With over 2.3 million incident cases diagnosed each year, breast cancer is the most common malignancy in women worldwide [1]. In western countries, 10-year survival after breast cancer is around 75% and increasing, due to widespread adoption of mammography screening and advancements in treatment efficacy [2–5]. Still, we found that among 10-year disease-free breast cancer survivors, there was a 17% cumulative incidence of breast cancer recurrence within 10-32 years after primary diagnosis [6]. This highlights the need for preventive strategies to reduce the risk of late breast cancer recurrence [7] (i.e. recurrence ≥10 years after primary diagnosis).

Aspirin is a nonsteroidal anti-inflammatory drug (NSAID) that irreversibly inhibits the cyclooxygenase enzymes COX-1 and COX-2 [8, 9]. Low-dose aspirin (75-150 mg per day) primarily inhibits COX-1, whereas high-dose aspirin (≥300 mg per day) inhibits both COX-1 and COX-2 [10]. COX-1 is ubiquitously expressed and important for vascular tone and platelet aggregation [9]. Platelets can bind tumour cells, facilitating immune evasion, angiogenesis, and promoting tumour cell survival and metastatic growth [11]. COX-2 is upregulated by inflammatory agents and overexpressed in up to 40% of breast tumours [9, 12, 13]. While COX-2 inhibition prevents the growth of mammary tumours in laboratory models [14, 15], observational and clinical studies of NSAIDs and selective COX-2 inhibitors have not provided evidence supporting their preventive effects on breast cancer recurrence [5, 16]. Furthermore, the recent Alliance A011502 (Aspirin after Breast Cancer, ABC) trial of high-dose aspirin was terminated early due to futility [17].

Observational studies investigating post-diagnostic aspirin use and breast cancer-specific mortality, recurrence, or contralateral breast cancer (CBC) have reported near-null associations [16, 18–23], but with maximum follow-up of 13 years [18]. Our previous research also showed little evidence to support an association between low-dose aspirin use and the risk of breast cancer recurrence or CBC [5, 24]. Still, pooled analyses of trial data suggested that any potential anti-cancer effects of aspirin could take 10 to 15 years to manifest [25, 26]. In addition, the CAPP2 trial, involving patients with Lynch syndrome, demonstrated a reduced incidence of colorectal cancer with aspirin use. This reduction first became evident about 5 years after the initiation of aspirin and persisted for over a decade [27]. Collectively, these findings indicate that the potential anti-cancer effects of aspirin may be most pronounced in long-term survivors.

In 2015, a large randomised clinical trial, Add-Aspirin, was launched to evaluate the effect of 5 years of daily use of 300 mg of aspirin on survival and risk of recurrence in breast, colorectal, gastroesophageal, and prostate cancers [28]. Although the results of this trial are highly awaited (recruitment continues until at least 2025), detecting long-term outcomes, such as late recurrence, in clinical trials is challenging [7]. This prompted us to conduct a large, population-based cohort study examining the association between low-dose aspirin use and risk of breast cancer recurrence, with up to 23 years of follow-up.

## Methods

### Study design and setting

Our nationwide cohort study was based on Danish population-based registries, taking advantage of the tax-funded universal healthcare system for all residents in Denmark. This includes access to primary care and hospital services, along with partial reimbursement for prescribed drugs [29]. We used the civil personal registration (CPR) number—a unique personal identifier assigned to all Danish residents at birth or upon immigration—to enable individual-level linkage across registries [30]. We linked data from the Danish Breast Cancer Group (DBCG) clinical database, the Danish National Patient Registry (DNPR), the National Prescription Registry, the Danish Civil Registration System, the Danish Pathology Registry and Patobank, the Danish Register of Causes of Death, and the Danish Cancer Registry [29–35]. In addition, we used data from a CBC database initiated for an earlier study [36] (see Supplementary files for details).

### Study population

We assembled a cohort of all women in Denmark who were at least 40 years old when diagnosed with incident stage I-III (i.e. non-distant metastatic) operable breast cancer, excluding in situ cancers, were registered in the DBCG clinical database, and were assigned a treatment protocol between January 1, 1996, and December 31, 2004. All women were followed according to contemporary DBCG guidelines [37].

### Aspirin exposure

From the Danish National Prescription Registry, we obtained data on low-dose aspirin prescriptions [Anatomical Therapeutic Chemical (ATC) = B01AC06; containing 75, 100 or 150 mg of acetylsalicylic acid per pill] filled by members of our study population.

We analysed aspirin exposure via a landmark approach [38] to avoid immortal time bias [39]. Assuming a package size of 100 pills and daily intake of one pill, we considered any use of aspirin as having filled at least two aspirin prescriptions between primary diagnosis and each landmark. To assess cumulative use of aspirin, a patient was considered exposed in any given year if she filled at least two prescriptions during that year. We used this approach for every year from primary diagnosis until study end. We then summed the total years of aspirin exposure from primary diagnosis to each landmark for all patients and categorised the results into 0 years (nonuser), 1–3 years, and >3 years of aspirin exposure. The cumulative aspirin exposure is depicted in Supplementary Fig. 1.

### Covariates

Age at diagnosis was categorised as 40–49, 50–59, 60–69 and ≥70 years in descriptive tables, but treated as a continuous variable in multivariable models. From the DBCG, we obtained information on patient, tumour, and treatment characteristics, including date of diagnosis, tumour size, histological grade, lymph node status, hormone receptor status, and treatments. We classified the tumour according to the Tumour, Node, Metastasis (TNM) and Union for International Cancer Control (UICC) classification systems [40]. Using the DNPR, we retrieved information on potentially confounding comorbidities and summarised these at primary diagnosis, and at the 5-, 10-, and 15-year landmarks using the Charlson Comorbidity Index score [41]. From the National Prescription Registry, we obtained information on potential confounding drugs, including statins, bisphosphonates, non-aspirin NSAIDs, angiotensin-converting enzyme (ACE) inhibitors, angiotensin receptor blockers (ARBs), digoxin, vitamin K anticoagulants, metformin, and overall hormone replacement therapy (HRT) (ATC codes are listed in Supplementary Table 1) [42–51]. A patient was considered exposed to any co-medication if she filled at least two prescriptions of the drug between diagnosis and each landmark. A patient was considered exposed to HRT if she had filled a prescription at any time before her breast cancer diagnosis.

### Outcomes

Information on breast cancer recurrences within the first 10 years after diagnosis was obtained from standard active surveillance activities of the DBCG [52]. For women who remained disease-free for 10 years (without recurrence, CBC or second cancer), we used a validated algorithm to detect late breast cancer recurrence (i.e. those occurring ≥10 years after primary diagnosis) [53]. We used the DNPR and the Danish Pathology Registry to further characterise algorithm-detected late breast cancer recurrences into local, regional, and distant recurrent cancer.

We examined both all-cause mortality and breast cancer-specific mortality as secondary outcomes. Information on causes of death was obtained from the Danish Register of Causes of Death. Breast cancer-specific death included deaths where breast cancer was listed either as the underlying cause of death or a contributing cause of death. Vital status and emigration were retrieved from the Civil Registration System and used to censor follow-up as appropriate.

### Statistical methods

We tabulated descriptive characteristics of the cohort at primary diagnosis according to low-dose aspirin use in the first year and at each landmark, i.e. 5, 10 and 15 years after primary diagnosis.

For the landmark analyses, we began follow-up at years 5, 10, and 15 after primary diagnosis, respectively. Patients who died, emigrated, or developed a recurrent or second cancer before the landmark were excluded. Using the Aalen-Johansen method, we plotted the cumulative incidence curves of recurrence, all-cause and breast cancer-specific mortality from 1 year after primary diagnosis, and from each landmark, according to aspirin use up until each of these time points. Further, we fitted crude and multivariable Cox proportional hazards regression models at each landmark to estimate hazard ratios (HRs) of recurrence or death, with corresponding 95% confidence intervals, comparing aspirin use with nonuse. Follow-up continued until recurrence, new primary cancer, death, emigration, or 31 December 2018. The adjusted models included age (continuous), menopausal status, comorbidities according to the Charlson Comorbidity Index (CCI) score, TNM stage and histologic grade, oestrogen receptor (ER) status, type of primary surgery, intended endocrine therapy, intended chemotherapy, pre-diagnostic exposure to HRT and post-diagnostic exposure to non-aspirin NSAIDs, statins, bisphosphonates, ACE inhibitors, ARBs, digoxin, metformin, and vitamin K anticoagulants. Given the low proportion of missing data, we conducted complete case analyses without applying imputations or other missing data techniques. We evaluated potential effect measure modification by stratifying analyses by primary tumour ER status, tumour stage, and grade.

We conducted several sensitivity analyses: 1) defining low-dose aspirin exposure as one or more prescriptions, or at least three prescriptions during 1 year; 2) a new-user design, restricted to patients with at least 5 years of prescription history, who never filled an aspirin prescription before primary breast cancer diagnosis.

The R code used for statistical analyses is available from the corresponding author upon request.

## Results

Among 20,509 breast cancer survivors, median follow-up was 12.6 years (interquartile range: 4.7–17.0), amounting to a total of 232,441 person-years of follow-up. Table 1 outlines characteristics of the study cohort at primary diagnosis according to low-dose aspirin use in the first year after breast cancer diagnosis. Women using low-dose aspirin were older and less likely to have undergone chemotherapy, but more likely to have received endocrine therapy, compared with nonusers. Additionally, low-dose aspirin users had more comorbidities and used more concomitant medications than nonusers. The clinical characteristics of the primary breast cancer—ER status, TNM stage, and histology—were similar among aspirin users and nonusers.

**Table 1:** ^a^Characteristics of 20,509 patients diagnosed with early-stage (non-distant metastatic) breast cancer during 1996–2004, according to aspirin use within the first year after diagnosis.

		Aspirin use within 1 year after breast cancer diagnosis	
	Total	Yes	No
	(*n* = 20,509)	(*n* = 850)	(*n* = 19,659)
Age (years)			
Median (IQR)	58 (51–66)	68 (62–72)	58 (51–65)
Age at primary breast cancer diagnosis (years)			
40–49	4108 (20%)	13 (2%)	4095 (21%)
50–59	7143 (35%)	133 (16%)	7010 (36%)
60–69	6349 (31%)	351 (41%)	5998 (31%)
≥70	2909 (14%)	353 (42%)	2556 (13%)
Menopausal status at primary diagnosis			
Pre-menopausal	<5500	<30	<5500
Post-menopausal	15,029 (73%)	825 (97%)	14,204 (72%)
Missing	<10	<10	<10
Charlson Comorbidity Index Score at primary diagnosis			
0	17,493 (85%)	419 (49%)	17,074 (87%)
1–2	2738 (13%)	374 (44%)	2364 (12%)
≥3	278 (1%)	57 (7%)	221 (1%)
Stage			
I	7804 (38%)	301 (35%)	7503 (38%)
II	8956 (44%)	393 (46%)	8563 (44%)
III	<3650	<200	<3500
Missing	<120	<20	<120
Grade			
I	7425 (36%)	328 (39%)	7097 (36%)
II	3881 (19%)	148 (17%)	3733 (19%)
III	3030 (15%)	123 (14%)	2907 (15%)
Not graded	0 (0%)	0 (0%)	0 (0%)
Missing	6173 (30.1%)	251 (29.5%)	5922 (30.1%)
Number of positive lymph nodes			
Negative	11,050 (54%)	473 (56%)	10,577 (54%)
1–3 positive nodes	5960 (29%)	236 (28%)	5724 (29%)
≥4 positive nodes	3481 (17%)	141 (17%)	3340 (17%)
Missing	18 (0.1%)	0 (0%)	18 (0.1%)
Tumour size			
≤20 mm	12,009 (59%)	451 (53%)	11,558 (59%)
>20 mm	<8500	<400	<8100
Missing	<10	<10	<10
ER status			
Negative	4318 (21%)	156 (18%)	4162 (21%)
Positive	15,379 (75%)	669 (79%)	14,710 (75%)
Missing	812 (4.0%)	25 (2.9%)	787 (4.0%)
Type of primary surgery			
Mastectomy + RT	4661 (23%)	153 (18%)	4508 (23%)
Mastectomy	8726 (43%)	466 (55%)	8260 (42%)
BCS + RT	7122 (35%)	231 (27%)	6891 (35%)
Allocated to adjuvant chemotherapy			
Yes	5616 (27%)	86 (10%)	5530 (28%)
No	14,893 (73%)	764 (90%)	14,129 (72%)
Allocated to endocrine therapy			
Yes	9785 (48%)	470 (55%)	9315 (47%)
No	10,724 (52%)	380 (45%)	10,344 (53%)
ACE inhibitors/Angiotensin receptor blockers^b^			
Yes	1884 (9%)	289 (34%)	1595 (8%)
No	18,625 (91%)	561 (66%)	18,064 (92%)
Statins			
Yes	736 (4%)	213 (25%)	523 (3%)
No	19,773 (96%)	637 (75%)	19,136 (97%)
Bisphosphonates			
Yes	187 (1%)	16 (2%)	171 (1%)
No	20,322 (99%)	834 (98%)	19,488 (99%)
Digoxin			
Yes	297 (1%)	63 (7%)	234 (1%)
No	20,212 (99%)	787 (93%)	19,425 (99%)
Non-aspirin NSAIDs			
Yes	4981 (24%)	277 (33%)	4704 (24%)
No	15,528 (76%)	573 (67%)	14,955 (76%)
Metformin			
Yes	212 (1%)	32 (4%)	180 (1%)
No	20,297 (99%)	818 (96%)	19,479 (99%)
Vitamin K anticoagulants			
Yes	242 (1%)	22 (3%)	220 (1%)
No	20,267 (99%)	828 (97%)	19,439 (99%)
Hormone replacement therapy			
Yes	9266 (45%)	413 (49%)	8853 (45%)
No	11,243 (55%)	437 (51%)	10,806 (55%)

*ACE* angiotensin-converting enzyme, *ARB* angiotensin receptor blockers, *BCS* breast-conserving surgery, *ER* oestrogen receptor, *IQR* interquartile range, *NSAID* nonsteroidal anti-inflammatory drugs, *RT* radiation therapy.

^a^In accordance with Danish data protection regulations, cell counts <5 and any cells that would allow back-calculation are reported in aggregate.

^b^For all confounding drugs, except hormone replacement therapy, numbers show use in the first year after breast cancer diagnosis. For hormone replacement therapy, numbers show ever use before breast cancer diagnosis.

Overall, 4118 women filled at least two low-dose aspirin prescriptions during follow-up. The median number of prescriptions was 17 (interquartile range: 7–33). During follow-up, 4527 patients were diagnosed with recurrent breast cancer; 1307 were late recurrences. In total, 3515 women died during follow-up; 1412 had breast cancer as the primary cause of death.

The 5-, 10-, and 15-year landmark cohorts comprised 15,128, 12,025 and 7983 disease-free survivors, respectively. At the 5-year landmark, 1475 women had at least 1 year of exposure to low-dose aspirin. The corresponding numbers at the 10- and 15-year landmarks were 2097 and 1734, respectively. Characteristics of the disease-free survivors at the landmarks are presented in Supplementary Tables 2–4.

**Fig. 1:**
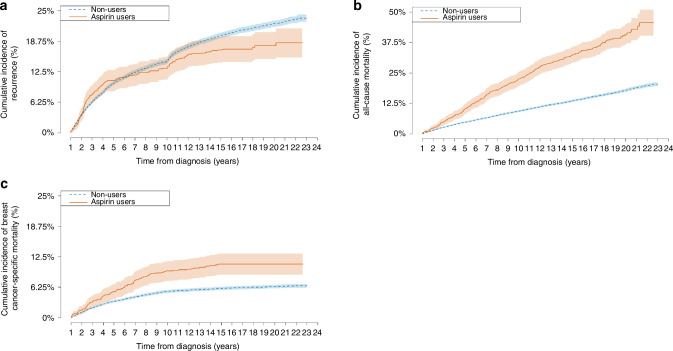
Cumulative incidences of outcomes from baseline. Cumulative incidence of (**a**) breast cancer recurrence, **b** all-cause mortality, and **c** breast cancer-specific mortality according to aspirin use in the first year following breast cancer diagnosis.

Among low-dose aspirin users, the cumulative incidence of breast cancer recurrence from 1 year after diagnosis was 10.7%, 13.2%, 17.0% and 17.8% at 5, 10, 15 and 20 years, respectively. Among nonusers, the corresponding numbers were 10.1%, 14.6%, 19.9% and 22.4% (Fig. 1a). The cumulative incidences of all-cause and breast cancer-specific mortality were higher among users compared with nonusers (Fig. 1b, c).

**Fig. 2:**
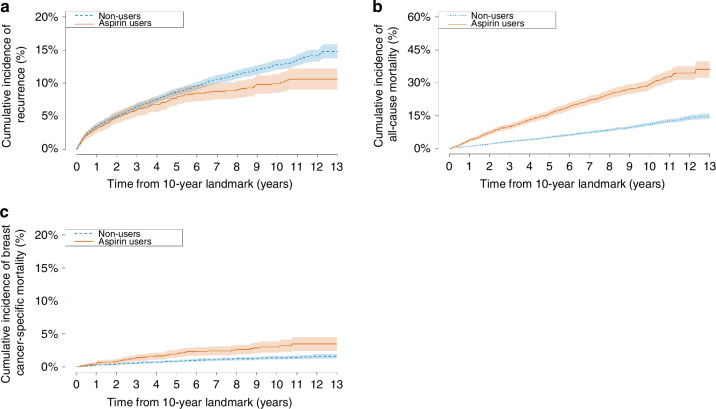
Cumulative incidences of outcomes at 10-year landmark. Cumulative incidence of (**a**) breast cancer recurrence, **b** all-cause mortality, and **c** breast cancer-specific mortality among patients alive and without a recurrent or second cancer (including contralateral breast cancer) at 10 years.

Among patients who were alive and free from recurrent or second cancer at 10 years post-diagnosis, the cumulative incidence of late recurrence among users was 7.8% at 15 years and 9.9% at 20 years post-diagnosis, while nonusers exhibited corresponding cumulative incidences of 8.6% and 12.7% (Fig. 2a). The cumulative incidences of all-cause and breast cancer-specific mortality were higher among low-dose aspirin users compared with nonusers (Fig. 2b, c).

**Fig. 3:**
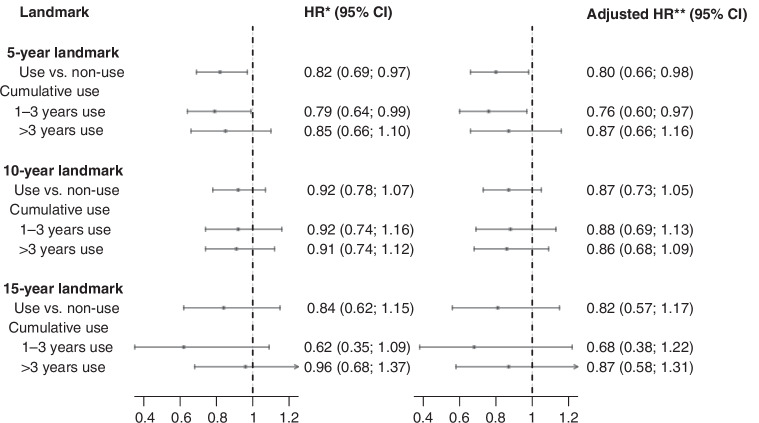
Hazard ratios (HRs) and 95% confidence intervals (CIs) for recurrence with aspirin use among patients in Denmark diagnosed with early-stage, non-distant metastatic breast cancer, alive and without recurrence or second cancer at years 5, 10 and 15 after primary diagnosis. CI confidence interval, HR hazard ratio. *Adjusted for age **Adjusted for age, calendar year of diagnosis, menopausal status, type of primary surgery, comorbidity status at primary diagnosis, oestrogen receptor status, stage, grade, chemotherapy, endocrine therapy, angiotensin-converting enzyme-inhibitors, angiotensin receptor blockers, statins, bisphosphonates, metformin, digoxin, hormone replacement therapy, non-aspirin NSAIDs, and vitamin K anticoagulants.

Any use of low-dose aspirin was associated with reduced HR of recurrence at all three landmarks (5-year: adjusted HR = 0.80 (95% CI = 0.66; 0.98), 10-year: HR = 0.87 (95% CI = 0.73; 1.05), and 15-year: HR = 0.82 (95% CI = 0.57; 1.17)) (Fig. 3). No evidence of effect modification was observed when analyses were stratified by stage, tumour grade or ER status (Supplementary Tables 5–7). Additionally, no apparent association was seen between longer duration of aspirin use and reduced risk of recurrence (Fig. 3).

**Fig. 4:**
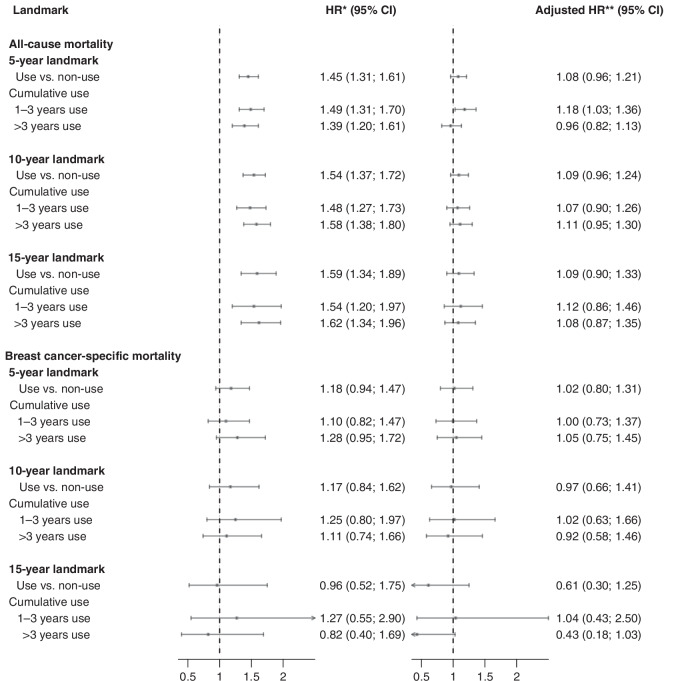
Hazard ratios (HRs) and 95% confidence intervals (CIs) for all-cause mortality and breast cancer-specific mortality with aspirin use among patients in Denmark diagnosed with early-stage, non-distant metastatic breast cancer, alive and without recurrence or second cancer at years 5, 10 and 15 after primary diagnosis. CI confidence interval, HR hazard ratio. *Adjusted for age **Adjusted for age, calendar year of diagnosis, menopausal status, type of primary surgery, comorbidity status at primary diagnosis, oestrogen receptor status, stage, grade, chemotherapy, endocrine therapy, angiotensin-converting enzyme-inhibitors, angiotensin receptor blockers, statins, bisphosphonates, metformin, digoxin, hormone replacement therapy, non-aspirin NSAIDs, and vitamin K anticoagulants.

Low-dose aspirin use was associated with increased mortality (5-year landmark cohort: adjusted HR = 1.08 (95% CI = 0.96; 1.21), 10-year: HR = 1.09 (95% CI = 0.96; 1.24), 15-year: HR = 1.09 (95% CI = 0.90; 1.33) (Fig. 3). For breast cancer-specific mortality, the associations attenuated (5-year: adjusted HR = 1.02 (95% CI = 0.80; 1.31), 10-year: HR = 0.97 (95% CI = 0.66; 1.41), 15-year: HR = 0.61 (95% CI = 0.30; 1.25)), albeit the estimates were imprecise (Fig. 4). Crude associations are presented in Supplementary Figs. 2 and 3.

Sensitivity analyses yielded results similar to those of the main analyses (Supplementary Tables 8 and 9).

## Discussion

Our findings suggest a reduced risk of breast cancer recurrence with the use of low-dose aspirin in women who survived at least 5 years after primary diagnosis. These findings remained consistent regardless of ER status, tumour grade and stage, or cumulative dose, or when applying a new-user design. We observed an increased absolute risk of all-cause and breast cancer-specific mortality among low-dose aspirin users. In adjusted analyses, the association attenuated for breast cancer-specific mortality, possibly due to deaths from other causes. Although we lacked information on the indication for low-dose aspirin use, this drug is predominantly prescribed in Denmark for cardiovascular disease prevention. The slight increase in mortality among aspirin users may be attributable to confounding by indication, which was supported by our finding that patients taking aspirin were older and had more comorbidities than nonusers. The attenuation in HRs from the crude to the adjusted model supports this explanation (Fig. 4, Supplementary Fig. 3). This could lead to death from other causes before experiencing recurrence, and might account for the observed reduced risks of recurrence among aspirin users.

Our observation of reduced risk of recurrence with low-dose aspirin use aligns with previous studies. A meta-analysis of 24 studies on aspirin use and survival among breast cancer patients reported a slight benefit of post-diagnostic aspirin use among survivors (HR of recurrence: 0.89, 95% CI 0.67–1.16) [54]. However, in contrast to our study, the meta-analysis also observed reduced risk of all-cause mortality with aspirin use compared with nonuse (HR of mortality: 0.87, 95% CI 0.71–1.07).

Reduced mortality among aspirin users was also reported in a Scottish study and two US-based studies [14, 55, 56]. However, methodological limitations of the Scottish study preclude further interpretation [5], and self-reported aspirin use in the US studies may have introduced exposure misclassification. Moreover, the US studies did not differentiate between low- and high-dose aspirin.

Our findings are important, particularly given the recent early termination of the ABC trial [17]. If the potential anti-cancer effect of aspirin is mediated by platelet inhibition, the higher 300 mg daily dose of aspirin applied in the ABC trial may have exceeded its potential anti-cancer mechanism of action. However, due to funding limitations, the trial was unable to evaluate the efficacy of lower doses of aspirin (i.e. ≤150 mg daily) [17]. Furthermore, the ABC trial lacked statistical precision to evaluate the effect of aspirin on different subtypes of breast cancer. A future pooling of the ABC trial results with those from the Add-Aspirin trial is planned to address these issues [17, 28]. In this regard, our study contributes to the existing literature by providing valuable insights into the long-term effects of low-dose aspirin, with extensive data on ER status, TNM stage and virtually complete follow-up.

Several considerations must be taken into account when interpreting our findings. First, while the data recorded in the DBCG clinical database generally have high validity, some regional differences in completeness may exist [52, 57]. A previous validation of the late recurrence algorithm demonstrated a positive predictive value of 85.7% (95% CI 77.5; 91.3), implying that up to 14% of patients registered with a late recurrence did not actually experience recurrent cancer. Although the sensitivity of the algorithm was 100%, the confidence intervals were broad, so some patients with recurrence may not have been correctly classified by the algorithm. In addition, patients with a high comorbidity burden might not readily associate new or altered symptoms with cancer recurrence. Still, cancer survivors with chronic diseases may have more frequent interaction with the healthcare system than those without, potentially leading to earlier and more complete detection of recurrent cancer in these patients. We attempted to account for this by adjusting for CCI at baseline and at the 10-year landmark. Second, we had no information on over-the-counter use of aspirin. However, the prevalence of over-the-counter use of low-dose aspirin misclassified as nonuse in the Danish registries is below 1% [58]. Thus, the impact of non-differential misclassification of true aspirin use is likely minimal in our study [58], and our estimates are likely to reflect actual aspirin use. Third, although we ascertained comprehensive data on redeemed prescriptions from the National Prescription Registry, we had no information on medication adherence. We therefore defined aspirin use as at least two prescriptions per year to minimise the influence of potential non-adherence. Fourth, in constructing and analysing our results at different landmarks, we created several selected populations of disease-free survivors. These groups are nested subsets of one another and are not directly comparable. Fifth, our study was conducted in a Danish population, which has predominantly European ancestry. As such, our findings may not be generalisable to all female populations. Ethnic variations in COX-2 gene expression and CYP2C9 enzyme activity could influence aspirin metabolism and treatment response [59–61]. Nevertheless, a Turkish study reported findings consistent with ours [62]. The ADD-Aspirin trial is currently ongoing with recruitment in both the United Kingdom and India. Thus, it may provide further insights into potential ethnic differences in treatment effects. Sixth, all analyses were performed as complete case analyses. For most variables, proportions of missing data were small. Last, although the Danish registries provided comprehensive data on multiple potential confounders, our results could still be prone to residual confounding, particularly from lifestyle factors.

Aspirin is an easily accessible, affordable drug with generally few side effects. As such, the potential of aspirin to reduce breast cancer recurrence risk has important public health implications. Our observed 10–20% decreased hazard rate of breast cancer recurrence in aspirin users may support a biological anti-cancer effect of aspirin. Still, the higher mortality among aspirin users indicates that our findings are likely influenced by confounding by indication and competing risks, whereby patients using low-dose aspirin might die from other causes before developing recurrent cancer. We await with anticipation the pooling of results from the ABC and Add-Aspirin trials.

## Supplementary information

Supplementaries

STROBE checklist

## Data Availability

In accordance with Danish privacy laws, the data underlying this article are not publicly available and cannot be shared. The analyses were performed on secure servers maintained by the Danish Health Data Authority. A detailed study protocol can be made available by contacting the corresponding author.
