# Supplementary material for: Association between low-dose aspirin use and breast cancer recurrence: a Danish nationwide cohort study with up to 23 years of follow-up
Source: Br J Cancer. 2025 Jul 22;133(6):865–73. doi: 10.1038/s41416-025-03112-3 (PMC12449462; doi:10.1038/s41416-025-03112-3)
Supplement: Supplementary file 1 — Supplementaries [file 41416_2025_3112_MOESM1_ESM.docx]

**Supplementary material
Title: Association between low-dose aspirin use and breast cancer recurrence: A Danish nationwide cohort study with 23 years of follow-up**

**Supplementary methods**

**Late breast cancer algorithm**

The algorithm incorporated information on diagnostic, therapeutic and procedural codes from the Danish National Patient Registry, and cancer diagnoses from the Danish Cancer Registry, the Danish Pathology Registry, the Danish National Patient Registry, the Danish Breast Cancer Group database, and a database on contralateral breast cancers.^1^

**Description of the databases**

Since 1977, the DBCG database has registered data on almost all women with an invasive breast cancer diagnosis in Denmark. The completeness has increased from 87% in 1986 to over 95% in 1997.^2^ The database contains data on tumor, treatment, and patient characteristics, which are prospectively collected by treating physicians. Up to approximately 2016, patients with operable breast cancer were followed up with biannual follow-up exams for the first five years, and annual exams up to ten years after primary diagnosis. Since 2016, patients on active treatment have been followed.^3^The follow-up exams include a clinical evaluation, and, if indicated, investigations to detect breast cancer recurrence such as x-ray, CT, or bone scans. The Danish National Patient Registry has collected administrative and clinical data on somatic inpatients since 1977, and since 1995, outpatients and emergency contacts.^4^ The data includes the CPR number, one primary diagnosis, one or more secondary diagnoses and data on diagnostic and surgical procedures. The National Prescription Registry has since 1995 recorded information on all prescription drugs dispensed at Danish pharmacies. Data includes the CPR number, the medications dispensed (classified according to the Anatomic Therapeutic Chemical system (ATC)), quantity, strength, and date of dispensing.^5^ The Danish Civil Registration system was established in 1968 and contains information on all Danish residents, including the date of birth, residence, emigration, and vital status.^6^ The Danish Pathology Registry was established in 1997 and has since recorded information on all pathological examinations performed in Denmark, including patient data, specimen type and quantity, and diagnoses based on the Danish Systemized Nomenclature of Medicine (SNOMED) codes.^7^ Since 1943, the Danish Cancer Registry has recorded data on all incident cancer cases in Denmark.^8^ Although the accuracy and completeness are high, information on contralateral breast cancers (CBC) is insufficient, and therefore a database on CBCs was established and included all CBCs diagnosed in Denmark between 1978 and 2013.^9^ At the time of writing, the database on CBCs was only updated until 2013. Consequently, we used the Danish Pathology Registry to identify CBCs occurring between 2014 and 2018. The Danish Cause of Death Registry has since 1970 registered underlying and contributory causes of death for all Danish citizens dying in Denmark.^10^

**Supplementary figures
Supplementary figure 1.** Aspirin use assessment.
To assess cumulative use of aspirin, a patient was considered exposed in any given year if she filled at least two prescriptions during that year. For example, if a patient did not fill any aspirin prescriptions during the first year after primary diagnosis, but filled three prescriptions between days 366 and 730, she was considered exposed to aspirin in year 2 but not in year 1. We used this approach for every year from primary diagnosis until study end. We then summed the total years of aspirin exposure from primary diagnosis to each landmark for all patients and categorized the results into 0 years (nonuser), 1-3 years, and >3 years of aspirin exposure. An arrow indicates a prescription was filled.


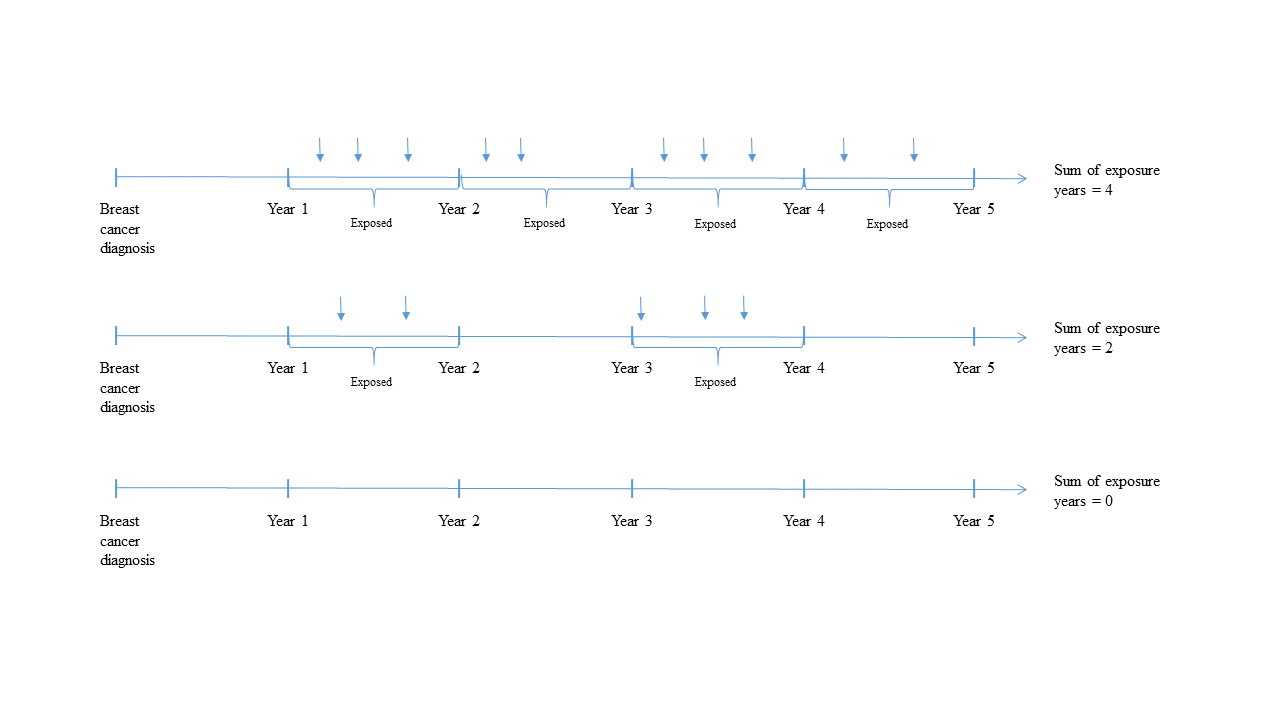


**Supplementary Figure 2.** Hazard ratios (HRs) and 95% confidence intervals (CIs) for recurrence with aspirin use among patients in Denmark diagnosed with early-stage, non-distant metastatic breast cancer, alive and without recurrence or second cancer at years 5, 10 and 15 after primary diagnosis.


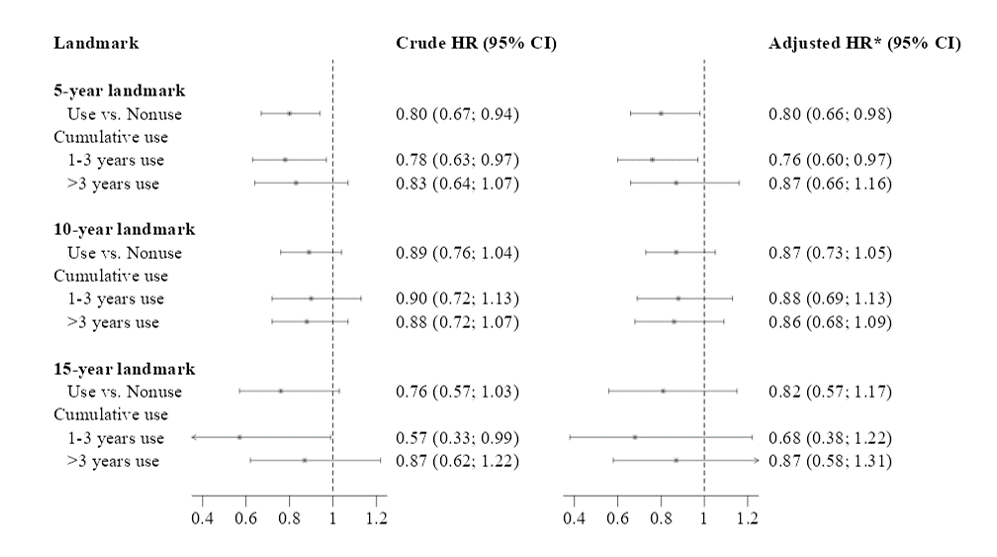


Abbreviations: CI, confidence interval; HR, hazard ratio
*Adjusted for age, calendar year of diagnosis, menopausal status, type of primary surgery, comorbidity status at primary diagnosis, estrogen receptor status, stage, grade, chemotherapy, endocrine therapy, angiotensin converting enzyme-inhibitors, angiotensin receptor blockers, statins, bisphosphonates, metformin, digoxin, hormone replacement therapy, non-aspirin NSAIDs, and vitamin K anticoagulants.

**Supplementary Figure 3.** Hazard ratios (HRs) and 95% confidence intervals (CIs) for all-cause mortality and breast cancer-specific mortality with aspirin use among patients in Denmark diagnosed with early-stage, non-distant metastatic breast cancer, alive and without recurrence or second cancer at years 5, 10 and 15 after primary diagnosis.

**
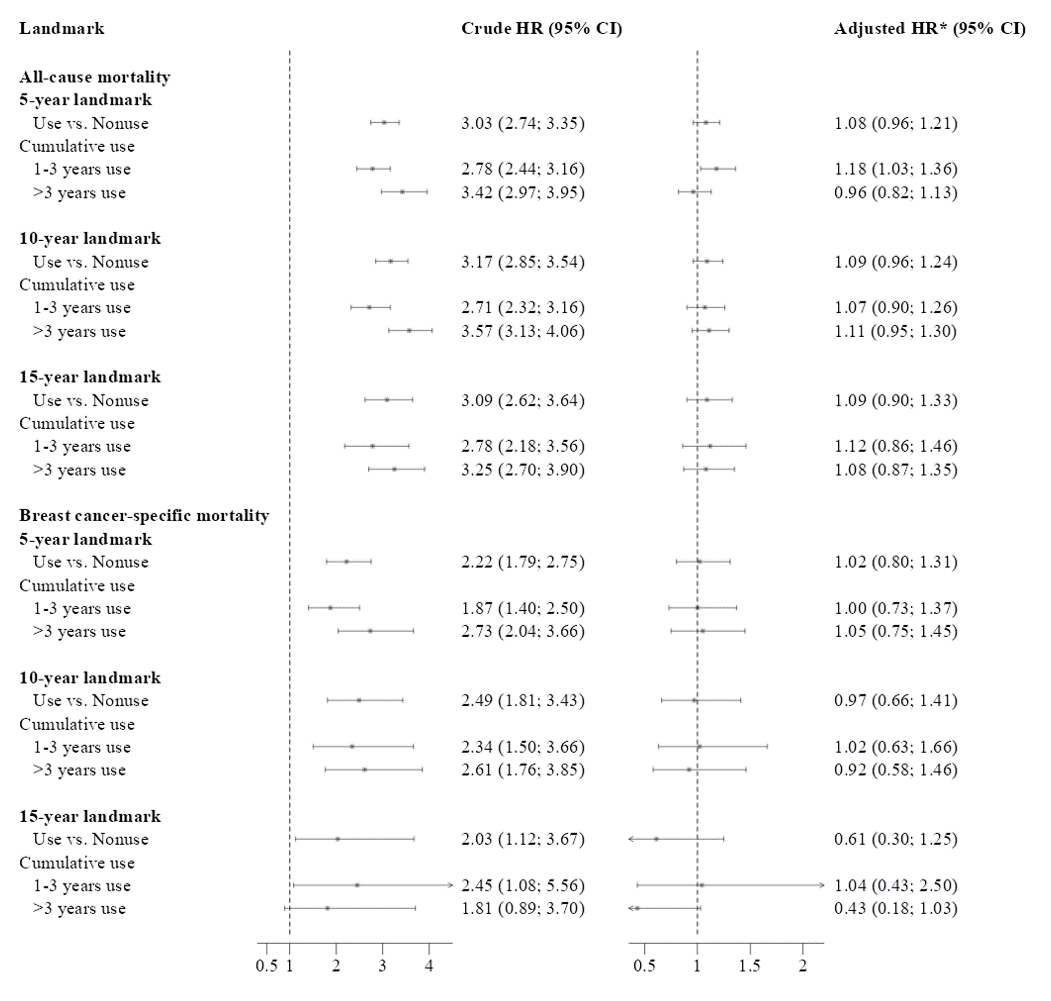
**

Abbreviations: CI, confidence interval; HR, hazard ratio
*Adjusted for age, calendar year of diagnosis, menopausal status, type of primary surgery, comorbidity status at primary diagnosis, estrogen receptor status, stage, grade, chemotherapy, endocrine therapy, angiotensin converting enzyme-inhibitors, angiotensin receptor blockers, statins, bisphosphonates, metformin, digoxin, hormone replacement therapy, non-aspirin NSAIDs, and vitamin K anticoagulants.

**Supplementary tables**

**Supplementary Table 1.** ATC codes.

| Drug class | ATC codes |
| --- | --- |
| Acetylsalicylic acid | B01AC06 |
| ACE inhibitors/ angiotensin receptor blockers | C09 |
| Statins | C10AA |
| Bisphosphonates | M05BA,  M05BB |
| Metformin (combinations included) | A10BA02, A10BD02, A10BD03, A10BD05, A10BD07, A10BD08, A10BD10, A10BD11, A10BD13, A10BD14, A10BD15, A10BD16, A10BD17, A10BD18, A10BD20, A10BD22, A10BD23, A10BD25, A10BD26, A10BD27 |
| Digoxin | C01AA |
| Hormone replacement therapy | G03C,  G03D,  G03F,  G03HB01 |
| Non-aspirin NSAIDs | M01A |
| Vitamin K anticoagulants | B01AA |

**Supplementary Table 2.** Descriptive characteristics of 15,128 patients alive and without a recurrence or second cancer at the 5-year landmark with an early-stage (non-distant metastatic) breast cancer diagnosis during 1996-2004, according to aspirin use between index date and the landmark.

|  | Aspirin use in the first 5 years following breast cancer diagnosis | | |
| --- | --- | --- | --- |
|  | Total | Yes | No |
|  | (n = 15,128) | (n = 1,475) | (n = 13,653) |
|  |  |  |  |
| Age |  |  |  |
| Median (Q1-Q3) | 57 ( 50 - 65) | 66 ( 60 - 71) | 57 ( 50 - 64) |
|  |  |  |  |
| Age at primary breast cancer diagnosis |  |  |  |
| 40-49 | 3221 (21 %) | 55 (4 %) | 3166 (23 %) |
| 50-59 | 5442 (36 %) | 302 (20 %) | 5140 (38 %) |
| 60-69 | 4568 (30 %) | 663 (45 %) | 3905 (29 %) |
| >=70 | 1897 (13 %) | 455 (31 %) | 1442 (11 %) |
|  |  |  |  |
| Menopausal status at primary breast cancer diagnosis |  |  |  |
| Pre-menopausal | <4400 | <100 | <4300 |
| Post-menopausal | 10786 (71 %) | 1386 (94 %) | 9400 (69 %) |
| Missing | <10 | <10 | <10 |
|  |  |  |  |
| Charlson Comorbidity Index Score at baseline |  |  |  |
| 0 | 13170 (87 %) | 950 (64 %) | 12220 (90 %) |
| 1-2 | 1814 (12 %) | 469 (32 %) | 1345 (10 %) |
| >=3 | 144 (1 %) | 56 (4 %) | 88 (1 %) |
|  |  |  |  |
| Charlson Comorbidity Index Score at the 5-year landmark |  |  |  |
| 0 | 11915 (79 %) | 635 (43 %) | 11280 (83 %) |
| 1-2 | 2845 (19 %) | 681 (46 %) | 2164 (16 %) |
| >=3 | 368 (2 %) | 159 (11 %) | 209 (2 %) |
|  |  |  |  |
| Stage |  |  |  |
| I | 6552 (43 %) | 617 (42 %) | 5935 (43 %) |
| II | 6714 (44 %) | 684 (46 %) | 6030 (44 %) |
| III | 1778 (12 %) | 163 (11 %) | 1615 (12 %) |
| Missing | 84 (0.6%) | 11 (0.7%) | 73 (0.5%) |
|  |  |  |  |
| Grade |  |  |  |
| I | 5452 (36 %) | 545 (37 %) | 4907 (36 %) |
| II | 2326 (15 %) | 225 (15 %) | 2101 (15 %) |
| III | 2347 (16 %) | 207 (14 %) | 2140 (16 %) |
| Not graded | 0 (0 %) | 0 (0 %) | 0 (0 %) |
| Missing | 5003 (33.1%) | 498 (33.8%) | 4505 (33.0%) |
|  |  |  |  |
| Number of positive lymph nodes |  |  |  |
| Negative | 9022 (60 %) | 879 (60 %) | 8143 (60 %) |
| 1-3 positive nodes | 4409 (29 %) | 437 (30 %) | 3972 (29 %) |
| >=4 positive nodes | <1700 | <200 | <1600 |
| Missing | <20 | <10 | <10 |
|  |  |  |  |
| Tumor size |  |  |  |
| <=20mm | 9668 (64 %) | 913 (62 %) | 8755 (64 %) |
| >20mm | <5500 | <600 | <4900 |
| Missing | <10 | <10 | <10 |
|  |  |  |  |
| ER status |  |  |  |
| Negative | 2714 (18 %) | 237 (16 %) | 2477 (18 %) |
| Positive | 11830 (78 %) | 1195 (81 %) | 10635 (78 %) |
| Missing | 584 (3.9%) | 43 (2.9%) | 541 (4.0%) |
|  |  |  |  |
| Type of primary surgery |  |  |  |
| Mastectomy + RT | 3003 (20 %) | 248 (17 %) | 2755 (20 %) |
| Mastectomy | 6314 (42 %) | 718 (49 %) | 5596 (41 %) |
| BCS + RT | 5811 (38 %) | 509 (35 %) | 5302 (39 %) |
|  |  |  |  |
| Allocated to adjuvant chemotherapy |  |  |  |
| Yes | 3969 (26 %) | 183 (12 %) | 3786 (28 %) |
| No | 11159 (74 %) | 1292 (88 %) | 9867 (72 %) |
|  |  |  |  |
| Allocated to endocrine therapy |  |  |  |
| Yes | 7204 (48 %) | 798 (54 %) | 6406 (47 %) |
| No | 7924 (52 %) | 677 (46 %) | 7247 (53 %) |
|  |  |  |  |
| Years of aspirin use |  |  |  |
| 0 years use | 13653 (90 %) | 0 (0 %) | 13653 (100 %) |
| 1-2 years use | 627 (4 %) | 627 (43 %) | 0 (0 %) |
| 3-5 years use | 848 (6 %) | 848 (57 %) | 0 (0 %) |
|  |  |  |  |
| ACE inhibitors/Angiotensin receptor blockers |  |  |  |
| Yes | 2631 (17 %) | 675 (46 %) | 1956 (14 %) |
| No | 12497 (83 %) | 800 (54 %) | 11697 (86 %) |
|  |  |  |  |
| Statins |  |  |  |
| Yes | 1812 (12 %) | 667 (45 %) | 1145 (8 %) |
| No | 13316 (88 %) | 808 (55 %) | 12508 (92 %) |
|  |  |  |  |
| Bisphosphonates |  |  |  |
| Yes | 656 (4 %) | 108 (7 %) | 548 (4 %) |
| No | 14472 (96 %) | 1367 (93 %) | 13105 (96 %) |
|  |  |  |  |
| Digoxin |  |  |  |
| Yes | 307 (2 %) | 118 (8 %) | 189 (1 %) |
| No | 14821 (98 %) | 1357 (92 %) | 13464 (99 %) |
|  |  |  |  |
| Non-aspirin NSAIDs |  |  |  |
| Yes | 6229 (41 %) | 765 (52 %) | 5464 (40 %) |
| No | 8899 (59 %) | 710 (48 %) | 8189 (60 %) |
|  |  |  |  |
| Metformin |  |  |  |
| Yes | 417 (3 %) | 145 (10 %) | 272 (2 %) |
| No | 14711 (97 %) | 1330 (90 %) | 13381 (98 %) |
|  |  |  |  |
| Vitamin K anticoagulants |  |  |  |
| Yes | 447 (3 %) | 107 (7 %) | 340 (2 %) |
| No | 14681 (97 %) | 1368 (93 %) | 13313 (98 %) |
|  |  |  |  |
| Hormone replacement therapy |  |  |  |
| Yes | 6280 (42 %) | 741 (50 %) | 5539 (41 %) |
| No | 8848 (58 %) | 734 (50 %) | 8114 (59 %) |

Abbreviations: ACE, angiotensin-converting enzyme; ARB, angiotensin receptor blockers; BCS, breast-conserving surgery; ER, estrogen receptor; IQR, interquartile range; NSAID, nonsteroidal anti-inflammatory drugs; RT, radiation therapy.
*Cell sizes <5 are reported in aggregate to reduce identifiability of individuals.
**For all confounding drugs, except hormone replacement therapy, numbers show use in the first year after breast cancer diagnosis. For hormone replacement therapy, numbers show ever use before breast cancer diagnosis.

**Supplementary Table 3.** Descriptive characteristics of 12,025 patients alive and without a recurrence or second cancer at the 10-year landmark with an early-stage (non-distant metastatic) breast cancer diagnosis during 1996-2004, according to aspirin use between index date and the landmark.

|  | Aspirin use in the first 10 years following breast cancer diagnosis | | |
| --- | --- | --- | --- |
|  | Total | Yes | No |
|  | (N=12,025) | (N=2,097) | (N=9,928) |
|  |  |  |  |
| Age |  |  |  |
| Median (Q1-Q3) | 57 ( 50 - 64) | 63 ( 57 - 69) | 55 ( 49 - 62) |
|  |  |  |  |
| Age at primary breast cancer diagnosis |  |  |  |
| 40-49 | 2791 (23 %) | 134 (6 %) | 2657 (27 %) |
| 50-59 | 4528 (38 %) | 567 (27 %) | 3961 (40 %) |
| 60-69 | 3491 (29 %) | 920 (44 %) | 2571 (26 %) |
| >=70 | 1215 (10 %) | 476 (23 %) | 739 (7 %) |
|  |  |  |  |
| Menopausal status at primary breast cancer diagnosis |  |  |  |
| Pre-menopausal | <3800 | <300 | <3600 |
| Post-menopausal | 8261 (69 %) | 1886 (90 %) | 6375 (64 %) |
| Missing | <10 | <10 | <10 |
|  |  |  |  |
| Charlson Comorbidity Index Score at baseline |  |  |  |
| 0 | 10662 (89 %) | 1582 (75 %) | 9080 (91 %) |
| 1-2 | 1289 (11 %) | 483 (23 %) | 806 (8 %) |
| >=3 | 74 (1 %) | 32 (2 %) | 42 (0 %) |
|  |  |  |  |
| Charlson Comorbidity Index Score at the 10 year landmark |  |  |  |
| 0 | 8689 (72 %) | 849 (40 %) | 7840 (79 %) |
| 1-2 | 2844 (24 %) | 968 (46 %) | 1876 (19 %) |
| >=3 | 492 (4 %) | 280 (13 %) | 212 (2 %) |
|  |  |  |  |
| Stage |  |  |  |
| I | 5428 (45 %) | 921 (44 %) | 4507 (45 %) |
| II | 5343 (44 %) | 971 (46 %) | 4372 (44 %) |
| III | 1182 (10 %) | 194 (9 %) | 988 (10 %) |
| Missing | 72 (0.6%) | 11 (0.5%) | 61 (0.6%) |
|  |  |  |  |
| Grade |  |  |  |
| I | 4283 (36 %) | 760 (36 %) | 3523 (35 %) |
| II | 1856 (15 %) | 315 (15 %) | 1541 (16 %) |
| III | 1858 (15 %) | 320 (15 %) | 1538 (15 %) |
| Not graded | 0 (0 %) | 0 (0 %) | 0 (0 %) |
| Missing | 4028 (33.5%) | 702 (33.5%) | 3326 (33.5%) |
|  |  |  |  |
| Number of positive lymph nodes |  |  |  |
| Negative | 7397 (62 %) | 1298 (62 %) | 6099 (61 %) |
| 1-3 positive nodes | 3502 (29 %) | 609 (29 %) | 2893 (29 %) |
| >=4 positive nodes | <1200 | <200 | <1000 |
| Missing | <10 | <10 | <10 |
|  |  |  |  |
| Tumor size |  |  |  |
| <=20mm | 7947 (66 %) | 1353 (65 %) | 6594 (66 %) |
| >20mm | <4100 | <800 | <3400 |
| Missing | <10 | <10 | <10 |
|  |  |  |  |
| ER status |  |  |  |
| Negative | 2225 (19 %) | 355 (17 %) | 1870 (19 %) |
| Positive | 9309 (77 %) | 1663 (79 %) | 7646 (77 %) |
| Missing | 491 (4.1%) | 79 (3.8%) | 412 (4.1%) |
|  |  |  |  |
| Type of primary surgery |  |  |  |
| Mastectomy + RT | 2286 (19 %) | 323 (15 %) | 1963 (20 %) |
| Mastectomy | 4897 (41 %) | 1020 (49 %) | 3877 (39 %) |
| BCS + RT | 4842 (40 %) | 754 (36 %) | 4088 (41 %) |
|  |  |  |  |
| Allocated to adjuvant chemotherapy |  |  |  |
| Yes | 3388 (28 %) | 318 (15 %) | 3070 (31 %) |
| No | 8637 (72 %) | 1779 (85 %) | 6858 (69 %) |
|  |  |  |  |
| Allocated to endocrine therapy |  |  |  |
| Yes | 5585 (46 %) | 1024 (49 %) | 4561 (46 %) |
| No | 6440 (54 %) | 1073 (51 %) | 5367 (54 %) |
|  |  |  |  |
| Years of aspirin use |  |  |  |
| 0 years use | 9928 (83 %) | 0 (0 %) | 9928 (100 %) |
| 1-2 years use | 623 (5 %) | 623 (30 %) | 0 (0 %) |
| 3-5 years use | 693 (6 %) | 693 (33 %) | 0 (0 %) |
| 5-7 years use | 333 (3 %) | 333 (16 %) | 0 (0 %) |
| > 7 years use | 448 (4 %) | 448 (21 %) | 0 (0 %) |
|  |  |  |  |
| ACE inhibitors/Angiotensin receptor blockers |  |  |  |
| Yes | 3499 (29 %) | 1221 (58 %) | 2278 (23 %) |
| No | 8526 (71 %) | 876 (42 %) | 7650 (77 %) |
|  |  |  |  |
| Statins |  |  |  |
| Yes | 3323 (28 %) | 1313 (63 %) | 2010 (20 %) |
| No | 8702 (72 %) | 784 (37 %) | 7918 (80 %) |
|  |  |  |  |
| Bisphosphonates |  |  |  |
| Yes | 1069 (9 %) | 255 (12 %) | 814 (8 %) |
| No | 10956 (91 %) | 1842 (88 %) | 9114 (92 %) |
|  |  |  |  |
| Digoxin |  |  |  |
| Yes | 318 (3 %) | 155 (7 %) | 163 (2 %) |
| No | 11707 (97 %) | 1942 (93 %) | 9765 (98 %) |
|  |  |  |  |
| Non-aspirin NSAIDs |  |  |  |
| Yes | 6953 (58 %) | 1413 (67 %) | 5540 (56 %) |
| No | 5072 (42 %) | 684 (33 %) | 4388 (44 %) |
|  |  |  |  |
| Metformin |  |  |  |
| Yes | 621 (5 %) | 272 (13 %) | 349 (4 %) |
| No | 11404 (95 %) | 1825 (87 %) | 9579 (96 %) |
|  |  |  |  |
| Vitamin K anticoagulants |  |  |  |
| Yes | 562 (5 %) | 211 (10 %) | 351 (4 %) |
| No | 11463 (95 %) | 1886 (90 %) | 9577 (96 %) |
|  |  |  |  |
| Hormone replacement therapy |  |  |  |
| Yes | 5000 (42 %) | 1043 (50 %) | 3957 (40 %) |
| No | 7025 (58 %) | 1054 (50 %) | 5971 (60 %) |
|  |  |  |  |

Abbreviations: ACE, angiotensin-converting enzyme; ARB, angiotensin receptor blockers; BCS, breast-conserving surgery; ER, estrogen receptor; IQR, interquartile range; NSAID, nonsteroidal anti-inflammatory drugs; RT, radiation therapy.
*Cell sizes <5 are reported in aggregate to reduce identifiability of individuals.
**For all confounding drugs, except hormone replacement therapy, numbers show use in the first year after breast cancer diagnosis. For hormone replacement therapy, numbers show ever use before breast cancer diagnosis.

**Supplementary Table 4.** Descriptive characteristics of 7,983 patients alive and without a recurrence or second cancer at the 15-year landmark with an early-stage (non-distant metastatic) breast cancer diagnosis during 1996-2004, according to aspirin use between index date and the landmark.

|  | Aspirin use in the first 15 years following breast cancer diagnosis | | |
| --- | --- | --- | --- |
|  | Total | Yes | No |
|  | (N=7,983) | (N=1,734) | (N=6,249) |
|  |  |  |  |
| Age |  |  |  |
| Median (Q1-Q3) | 55 ( 49 - 62) | 61 ( 55 - 67) | 54 ( 48 - 60) |
|  |  |  |  |
| Age at primary breast cancer diagnosis |  |  |  |
| 40-49 | 2045 (26 %) | 165 (10 %) | 1880 (30 %) |
| 50-59 | 3198 (40 %) | 568 (33 %) | 2630 (42 %) |
| 60-69 | 2188 (27 %) | 749 (43 %) | 1439 (23 %) |
| >=70 | 552 (7 %) | 252 (15 %) | 300 (5 %) |
|  |  |  |  |
| Menopausal status at primary breast cancer diagnosis |  |  |  |
| Pre-menopausal | <2800 | <250 | <2500 |
| Post-menopausal | 5244 (66 %) | 1491 (86 %) | 3753 (60 %) |
| Missing | <10 | <10 | <10 |
|  |  |  |  |
| Charlson Comorbidity Index Score at baseline |  |  |  |
| 0 | 7264 (91 %) | 1418 (82 %) | 5846 (94 %) |
| 1-2 | 691 (9 %) | 300 (17 %) | 391 (6 %) |
| >=3 | 28 (0 %) | 16 (1 %) | 12 (0 %) |
|  |  |  |  |
| Charlson Comorbidity Index Score at the 15 year landmark |  |  |  |
| 0 | 5328 (67 %) | 644 (37 %) | 4684 (75 %) |
| 1-2 | 2215 (28 %) | 816 (47 %) | 1399 (22 %) |
| >=3 | 440 (6 %) | 274 (16 %) | 166 (3 %) |
|  |  |  |  |
| Stage |  |  |  |
| I | 3762 (47 %) | 824 (48 %) | 2938 (47 %) |
| II | 3488 (44 %) | 768 (44 %) | 2720 (44 %) |
| III | 681 (9 %) | 133 (8 %) | 548 (9 %) |
| Missing | 52 (0.7%) | 9 (0.5%) | 43 (0.7%) |
|  |  |  |  |
| Grade |  |  |  |
| I | 2738 (34 %) | 577 (33 %) | 2161 (35 %) |
| II | 1261 (16 %) | 269 (16 %) | 992 (16 %) |
| III | 1301 (16 %) | 286 (16 %) | 1015 (16 %) |
| Not graded | 0 (0 %) | 0 (0 %) | 0 (0 %) |
| Missing | 2683 (33.6%) | 602 (34.7%) | 2081 (33.3%) |
|  |  |  |  |
| Number of positive lymph nodes |  |  |  |
| Negative | 5085 (64 %) | 1132 (65 %) | 3953 (63 %) |
| 1-3 positive nodes | 2251 (28 %) | 470 (27 %) | 1781 (29 %) |
| >=4 positive nodes | <700 | <150 | <550 |
| Missing | <10 | <10 | <10 |
|  |  |  |  |
| Tumor size |  |  |  |
| <=20mm | 5361 (67 %) | 1157 (67 %) | 4204 (67 %) |
| >20mm | 2622 (33 %) | 577 (33 %) | 2045 (33 %) |
|  |  |  |  |
| ER status |  |  |  |
| Negative | 1636 (20 %) | 336 (19 %) | 1300 (21 %) |
| Positive | 5993 (75 %) | 1329 (77 %) | 4664 (75 %) |
| Missing | 354 (4.4%) | 69 (4.0%) | 285 (4.6%) |
|  |  |  |  |
| Type of primary surgery |  |  |  |
| Mastectomy + RT | 1494 (19 %) | 263 (15 %) | 1231 (20 %) |
| Mastectomy | 3343 (42 %) | 850 (49 %) | 2493 (40 %) |
| BCS + RT | 3146 (39 %) | 621 (36 %) | 2525 (40 %) |
|  |  |  |  |
| Allocated to adjuvant chemotherapy |  |  |  |
| Yes | 2388 (30 %) | 295 (17 %) | 2093 (33 %) |
| No | 5595 (70 %) | 1439 (83 %) | 4156 (67 %) |
|  |  |  |  |
| Allocated to endocrine therapy |  |  |  |
| Yes | 3379 (42 %) | 730 (42 %) | 2649 (42 %) |
| No | 4604 (58 %) | 1004 (58 %) | 3600 (58 %) |
|  |  |  |  |
| Years of aspirin use |  |  |  |
| 0 years use | 6249 (78 %) | 0 (0 %) | 6249 (100 %) |
| 1-2 years use | 404 (5 %) | 404 (23 %) | 0 (0 %) |
| 3-5 years use | 462 (6 %) | 462 (27 %) | 0 (0 %) |
| 5-7 years use | 232 (3 %) | 232 (13 %) | 0 (0 %) |
| > 7 years use | 636 (8 %) | 636 (37 %) | 0 (0 %) |
|  |  |  |  |
| ACE inhibitors/Angiotensin receptor blockers |  |  |  |
| Yes | 2935 (37 %) | 1094 (63 %) | 1841 (29 %) |
| No | 5048 (63 %) | 640 (37 %) | 4408 (71 %) |
|  |  |  |  |
| Statins |  |  |  |
| Yes | 2991 (37 %) | 1237 (71 %) | 1754 (28 %) |
| No | 4992 (63 %) | 497 (29 %) | 4495 (72 %) |
|  |  |  |  |
| Bisphosphonates |  |  |  |
| Yes | 1089 (14 %) | 307 (18 %) | 782 (13 %) |
| No | 6894 (86 %) | 1427 (82 %) | 5467 (87 %) |
|  |  |  |  |
| Digoxin |  |  |  |
| Yes | 247 (3 %) | 137 (8 %) | 110 (2 %) |
| No | 7736 (97 %) | 1597 (92 %) | 6139 (98 %) |
|  |  |  |  |
| Non-aspirin NSAIDs |  |  |  |
| Yes | 5323 (67 %) | 1268 (73 %) | 4055 (65 %) |
| No | 2660 (33 %) | 466 (27 %) | 2194 (35 %) |
|  |  |  |  |
| Metformin |  |  |  |
| Yes | 600 (8 %) | 289 (17 %) | 311 (5 %) |
| No | 7383 (92 %) | 1445 (83 %) | 5938 (95 %) |
|  |  |  |  |
| Vitamin K anticoagulants |  |  |  |
| Yes | 462 (6 %) | 214 (12 %) | 248 (4 %) |
| No | 7521 (94 %) | 1520 (88 %) | 6001 (96 %) |
|  |  |  |  |
| Hormone replacement therapy |  |  |  |
| Yes | 3264 (41 %) | 834 (48 %) | 2430 (39 %) |
| No | 4719 (59 %) | 900 (52 %) | 3819 (61 %) |

Abbreviations: ACE, angiotensin-converting enzyme; ARB, angiotensin receptor blockers; BCS, breast-conserving surgery; ER, estrogen receptor; IQR, interquartile range; NSAID, nonsteroidal anti-inflammatory drugs; RT, radiation therapy.
*Cell sizes <5 are reported in aggregate to reduce identifiability of individuals.
**For all confounding drugs, except hormone replacement therapy, numbers show use in the first year after breast cancer diagnosis. For hormone replacement therapy, numbers show ever use before breast cancer diagnosis.

**Supplementary Table 5.** Hazard ratios (HRs) and 95% confidence intervals (CIs) of recurrence associated with aspirin use among patients diagnosed with an early-stage non-distant metastatic breast cancer in Denmark, alive and without a recurrence or second cancer at years 5, 10 and 15 after primary diagnosis. Results are stratified by stage.

| Landmark | Stage I | Stage II | Stage III |
| --- | --- | --- | --- |
| 5 year landmark |  |  |  |
| Nonusers | Ref. | Ref. | Ref. |
| Aspirin users | 0.80 (0.68; 0.95) | 0.80 (0.68; 0.95) | 0.81 (0.69; 0.96) |
|  |  |  |  |
| 10 year landmark |  |  |  |
| Nonusers | Ref. | Ref. | Ref. |
| Aspirin users | 0.89 (0.76; 1.04) | 0.89 (0.77; 1.04) | 0.90 (0.77; 1.05) |
|  |  |  |  |
| 15 year landmark |  |  |  |
| Nonusers | Ref. | Ref. | Ref. |
| Aspirin users | 0.78 (0.57; 1.05) | 0.78 (0.57; 1.05) | 0.78 (0.58; 1.06) |

**Supplementary Table 6.** Hazard ratios (HRs) and 95% confidence intervals (CIs) of recurrence associated with aspirin use among patients diagnosed with an early-stage non-distant metastatic breast cancer in Denmark, alive and without a recurrence or second cancer at years 5, 10 and 15 after primary diagnosis. Results are stratified by grade.

| Landmark | Grade I | Grade II | Grade III |
| --- | --- | --- | --- |
| 5 year landmark |  |  |  |
| Nonusers | Ref. | Ref. | Ref. |
| Aspirin users | 0.77 (0.65; 0.92) | 0.77 (0.65; 0.92) | 0.77 (0.65; 0.92) |
|  |  |  |  |
| 10 year landmark |  |  |  |
| Nonusers | Ref. | Ref. | Ref. |
| Aspirin users | 0.88 (0.75; 1.02) | 0.88 (0.75; 1.03) | 0.88 (0.75; 1.02) |
|  |  |  |  |
| 15 year landmark |  |  |  |
| Nonusers | Ref. | Ref. | Ref. |
| Aspirin users | 0.75 (0.55; 1.02) | 0.75 (0.55; 1.02) | 0.75 (0.55; 1.02) |

**Supplementary Table 7.** Hazard ratios (HRs) and 95% confidence intervals (CIs) of recurrence associated with aspirin use among patients diagnosed with an early-stage non-distant metastatic breast cancer in Denmark, alive and without a recurrence or second cancer at years 5, 10 and 15 after primary diagnosis. Results are stratified by ER status.

| Landmark | ER positive | ER negative |
| --- | --- | --- |
| 5 year landmark |  |  |
| Nonusers | Ref. | Ref. |
| Aspirin users | 0.78 (0.66; 0.93) | 0.78 (0.66; 0.93) |
|  |  |  |
| 10 year landmark |  |  |
| Nonusers | Ref. | Ref. |
| Aspirin users | 0.85 (0.72; 0.99) | 0.85 (0.72; 0.99) |
|  |  |  |
| 15 year landmark |  |  |
| Nonusers | Ref. | Ref. |
| Aspirin users | 0.74 (0.55; 1.01) | 0.74 (0.55; 1.01) |

**Supplementary Table 8.** Hazard ratios (HRs) and 95% confidence intervals (CIs) of recurrence associated with aspirin use among patients diagnosed with an early-stage non-distant metastatic breast cancer in Denmark, alive and without a recurrence or second cancer at years 5, 10 and 15 after primary diagnosis. Sensitivity analysis. Use of aspirin changed to to ≥1 or ≥3 aspirin prescriptions in a year during follow-up.

| Use defined as ≥1 aspirin prescription | | | | |
| --- | --- | --- | --- | --- |
|  | | | | |
| Landmark | | **Crude HR (95%CI)** | | **Adjusted HR* (95%CI)** |
| 5 year landmark | |  | |  |
| Nonusers | | Ref. | | Ref. |
| Aspirin users | | 0.76 (0.65; 0.89) | | 0.76 (0.64; 0.91) |
|  | |  | |  |
| 10 year landmark | |  | |  |
| Nonusers | | Ref. | | Ref. |
| Aspirin users | | 0.91 (0.79; 1.04) | | 0.91 (0.77; 1.07) |
|  | |  | |  |
| 15 year landmark | |  | |  |
| Nonusers | | Ref. | | Ref. |
| Aspirin users | | 0.92 (0.71; 1.20) | | 1.00 (0.73; 1.37) |
|  | |  | |  |
| Use defined as ≥3 aspirin prescriptions | | | | |
|  | | | | |
| Landmark | **Crude HR (95%CI)** | | **Adjusted HR* (95%CI)** | |
| 5 year landmark |  | |  | |
| Nonusers | Ref. | | Ref. | |
| Aspirin users | 0.81 (0.69; 0.99) | | 0.84 (0.69; 1.03) | |
|  |  | |  | |
| 10 year landmark |  | |  | |
| Nonusers | Ref. | | Ref. | |
| Aspirin users | 0.88 (0.75; 1.03) | | 0.86 (0.71; 1.04) | |
|  |  | |  | |
| 15 year landmark |  | |  | |
| Nonusers | Ref. | | Ref. | |
| Aspirin users | 0.79 (0.58; 1.09) | | 0.84 (0.58; 1.22) | |

Abbreviations: CI, confidence interval; HR, hazard ratio
*Adjusted for age, calendar year of diagnosis, menopausal status, type of primary surgery, comorbidity status at primary diagnosis, estrogen receptor status, stage, grade, chemotherapy, endocrine therapy, angiotensin converting enzyme-inhibitors, angiotensin receptor blockers, statins, bisphosphonates, metformin, digoxin, hormone replacement therapy, non-aspirin NSAIDs, vitamin K anticoagulants

**Supplementary Table 9.** Hazard ratios (HRs) and 95% confidence intervals (CIs) of recurrence associated with aspirin use among patients diagnosed with an early-stage non-distant metastatic breast cancer in Denmark, alive and without a recurrence or second cancer at years 5, 10 and 15 after primary diagnosis. Sensitivity analysis. New user design.

| Landmark | Crude | Adjusted HR* (95%CI) |
| --- | --- | --- |
| 5 year landmark |  |  |
| Nonusers | Ref. | Ref. |
| Aspirin users | 0.77 (0.59; 0.99) | 0.80 (0.60; 1.07) |
|  |  |  |
| 10 year landmark |  |  |
| Nonusers | Ref. | Ref. |
| Aspirin users | 0.90 (0.72; 1.04) | 0.98 (0.76; 1.28) |
|  |  |  |
| 15 year landmark |  |  |
| Nonusers | Ref. | Ref. |
| Aspirin users | 0.94 (0.55; 1.61) | 1.08 (0.57; 2.04) |

Abbreviations: CI, confidence interval; HR, hazard ratio

*Adjusted for age, calendar year of diagnosis, menopausal status, type of primary surgery, comorbidity status at primary diagnosis, estrogen receptor status, stage, grade, chemotherapy, endocrine therapy, angiotensin converting enzyme-inhibitors, angiotensin receptor blockers, statins, bisphosphonates, metformin, digoxin, hormone replacement therapy, non-aspirin NSAIDs, vitamin K anticoagulants

**References**

1. Pedersen RN, Öztürk B, Mellemkjær L, et al. Validation of an Algorithm to Ascertain Late Breast Cancer Recurrence Using Danish Medical Registries. *Clin Epidemiol*. 2020;12:1083. doi:10.2147/CLEP.S269962

2. Blichert-Toft M, Christiansen P, Mouridsen HT. Danish Breast Cancer Cooperative Group – DBCG: History, organization, and status of scientific achievements at 30-year anniversary. *Acta Oncol*. 2008;47(4):497-505. doi:10.1080/02841860802068615

3. Saltbæk L, Horsboel TA, Offersen BV, et al. Patterns in detection of recurrence among patients treated for breast cancer. *Breast Cancer Res Treat*. 2020;184(2):365-373. doi:10.1007/s10549-020-05847-4

4. Schmidt M, Schmidt SAJ, Sandegaard JL, Ehrenstein V, Pedersen L, Sørensen HT. The Danish National Patient Registry: a review of content, data quality, and research potential. *Clin Epidemiol*. 2015;7:449-490. doi:10.2147/CLEP.S91125

5. Kildemoes HW, Sørensen HT, Hallas J. The Danish National Prescription Registry. *Scand J Public Health*. 2011;39(7 Suppl):38-41. doi:10.1177/1403494810394717

6. Schmidt M, Pedersen L, Sørensen HT. The Danish Civil Registration System as a tool in epidemiology. *Eur J Epidemiol*. 2014;29(8):541-549. doi:10.1007/s10654-014-9930-3

7. Erichsen R, Lash TL, Hamilton-Dutoit SJ, Bjerregaard B, Vyberg M, Pedersen L. Existing data sources for clinical epidemiology: the Danish National Pathology Registry and Data Bank. *Clin Epidemiol*. 2010;2:51-56. doi:10.2147/CLEP.S9908

8. Gjerstorff ML. The Danish Cancer Registry. *Scand J Public Health*. 2011;39(7 Suppl):42-45. doi:10.1177/1403494810393562

9. Langballe R, Frederiksen K, Jensen MB, et al. Mortality after contralateral breast cancer in Denmark. *Breast Cancer Res Treat*. 2018;171(2):489-499. doi:10.1007/s10549-018-4846-3

10. Helweg-Larsen K. The Danish Register of Causes of Death. *Scand J Public Health*. 2011;39(7 Suppl):26-29. doi:10.1177/1403494811399958
